# Predictors of uncontrolled hypertension and antihypertensive medication nonadherence

**DOI:** 10.4103/0975-3583.74263

**Published:** 2010

**Authors:** Manuel Morgado, Sandra Rolo, Ana Filipa Macedo, Luísa Pereira, Miguel Castelo-Branco

**Affiliations:** 1 *Health Sciences Research Centre, University of Beira Interior, Av. Infante D. Henrique 6200-506, Portugal*; 2 *Hospital Centre of Cova da Beira, E.P.E., Quinta do Alvito, 6200-251, Portugal*; 3 *Mathematics Department, University of Beira Interior, Rua Marquês d’Ávila e Bolama, 6201-001 Covilhã, Portugal*

**Keywords:** Antihypertensives, blood pressure control, hypertension, medication adherence, Portugal

## Abstract

**Background:**

Although hypertension is, in most cases, a controllable major risk factor in the development of cardiovascular disease, studies have demonstrated that hypertension remains poorly controlled in Portugal. Our aim was to evaluate the covariates associated with poor blood pressure (BP) control in a Portuguese hypertensive population.

**Patients and Results:**

We conducted a cross-sectional survey in a hospital hypertension outpatient clinic, located in the Eastern Central Region of Portugal. Patients attending the clinic from July to September 2009 were asked to participate in a structured interview including medication adherence and knowledge about hypertension. Eligible participants were all adults aged 18 or over with an established diagnosis of arterial hypertension and had been on antihypertensive drug treatment for at least 6 months. Exclusion criteria were dementia, pregnancy, and breastfeeding. Detailed clinical information was prospectively obtained from medical records. A total of 197 patients meeting the inclusion criteria and consenting to participate completed the interview. Of these, only 33.0% had their BP controlled according to the JNC 7 guidelines. Logistic regression analysis revealed three independent predictors of poor BP control: living alone (OR = 5.3, *P* = 0.004), medication nonadherence (OR = 4.8, *P* < 0.001), and diabetes (OR = 4.4, *P* = 0.011). Predictors of medication nonadherence were: unawareness of target BP values (OR = 3.7, *P* < 0.001), a report of drug side effects (OR = 3.7, *P* = 0.002), lack of BP monitoring (OR = 2.5, *P* = 0.015) and unawareness of medication indications (OR = 2.4, *P* = 0.021), and of hypertension risks (OR = 2.1, *P* = 0.026).

**Conclusions:**

Poor medication adherence, lack of information about hypertension, and side effects should be considered as possible underlying causes of uncontrolled BP and must be addressed in any intervention aimed to improve BP control.

## INTRODUCTION

Hypertension is a major risk factor in the development of cardiovascular disease and one of the most important public health problems in Portugal affecting over 3 million Portuguese adults (about 30% of the Portuguese population).[1] In well-conducted clinical trials, numerous drugs and combination therapies have demonstrated their ability to reduce blood pressure (BP), with rates of BP control ranging from 45% to 66%.[2,3] However, in the clinical practice, control rates of high BP are expected to be lower than those observed in the high motivated, closely controlled, and monitored patient population of the clinical trials. In a recently published survey,[1] only 28.9% Portuguese treated hypertensives had their BP controlled (<140/90 mmHg). This figure is even lower in the Central Region of Portugal, where only 26.1% of the total number of treated hypertensives have their BP controlled.[1] One of the major drawbacks of this Portuguese survey relies on the definition of controlled hypertension, which was considered as the mean systolic BP <140 mmHg and diastolic BP of <90 mmHg. This definition does not take into account the seventh report of the Joint National Committee on the Prevention, Detection, Evaluation, and Treatment of High Blood Pressure (JNC 7), according to which for hypertensive patients who also have diabetes or chronic kidney disease (CKD) target levels of BP are even lower (<130/80 mmHg).

The Eastern Central Region of Portugal possesses a university teaching hospital at Covilhã, named Cova da Beira Hospital Centre, with an important secondary care hypertension/dyslipidemia outpatient clinic, which serves a significant hypertensive population of the District of Castelo Branco. To better understand the unsatisfactory levels of BP control of this hypertensive population and focus efforts on improving them, it would be useful to know the extent to which hypertensive patients with different risks for vascular complications are not satisfactorily controlled and to identify some possible factors that may be involved in inadequate BP control. Accordingly, we conducted a cross-sectional study to evaluate the level of BP control in hypertensive patients attending the mentioned hospital hypertension/dyslipidemia outpatient clinic and to recognize some factors that may underlie insufficient BP control and poor medication adherence.

## PATIENTS AND METHODS

### Settings and study design

In July–September 2009, we conducted a cross-sectional survey in a secondary care hypertension/dyslipidemia clinic in the university teaching hospital of Cova da Beira Hospital Centre, Covilhã, District of Castelo Branco, located in the Eastern Central Region of Portugal. This outpatient clinic is one of the most important clinics of this region in Portugal in the field of hypertension/dyslipidemia and serves a significant hypertensive population of Covilhã Area, with a population of 35 thousand inhabitants.

### Study population

All hypertensive patients attending the medical clinic during that period were asked to complete the structured interview. Patients were asked to complete a structured questionnaire on demography, medication adherence, and knowledge about target BP values, hypertension risks, indications of antihypertensive medication, and the presence of drug side effects. Subjects were also asked whether they used a home BP monitoring device and/or whether they measure their BP regularly. The study was approved by the Institutional Ethics Committee for the use of humans in research, and written informed consent was obtained from all participants before their enrolment in the study.

Eligible participants were all adults aged 18 or over with an established medical diagnosis of arterial hypertension (BP measurements in the clinic of systolic BP (SBP) ≥140 mmHg and/or diastolic BP (DBP) ≥90 mm Hg). Furthermore, all included patients had been on established antihypertensive drug treatment for at least 6 months. Exclusion criteria were dementia, pregnancy, and breastfeeding.

### Blood Pressure measurements

BP was measured in a seated position after a 5-min rest period, using a mercury sphygmomanometer or semi-automatic device, the mean of two consecutive measurements being recorded. According to the JNC 7 guidelines, hypertensive patients without diabetes and CKD with BP <140/90 mmHg were considered to have their BP controlled. For hypertensive patients with diabetes or CKD, BP control was defined as BP measurements <130/80 mmHg.

### Medication adherence, hypertension knowledge, and management evaluation

Assessment of antihypertensive medication adherence was determined using the instrument validated by Morisky *et al*.[4,5] Poor medication adherence was defined as answering yes to three or more of five questions. We also evaluated patient knowledge of target BP values and of the potential impact of hypertension on the morbidity and mortality associated with stroke, cardiac disease, and kidney disease. Patients were considered knowledgeable of target BP values if they knew both target BP figures (<140/90 mmHg for hypertensive patients without diabetes and CKD and <130/<80 mmHg for hypertensive patients with diabetes or CKD). They were considered knowledgeable of the negative impacts of hypertension to health if they mentioned at least two potential major negative consequences of uncontrolled hypertension. It was considered that BP was measured regularly if values were recorded at least once a month.

### Clinical parameters

Clinical data for this study, including BP measures, medications prescribed, and medical problems, were prospectively obtained from the Hospital Electronic Medical Records (HEMR) database. The HEMR database of Cova da Beira Hospital Centre is comprised of detailed patient-level clinical and administrative information of all patients that have used this hospital at least once. Available information includes patient demographics, medical problems, various measures of physiological status, and medications prescribed. This database is authorized by the Portugal Department of Health, the government department responsible for public health issues, and patient data confidentiality was ensured.

### Statistical analysis

Demographic variables, clinical data, and BP values of hypertensive patients included in the study, as well as prescribing metrics were examined on a descriptive basis and expressed as the mean ± SD, frequency, and percentages. To test for differences between categorical variables χ^2^ -test were used. Multivariate analyses were conducted using logistic regressions with the forward likelihood ratio (Forward:LR) selection algorithm. All statistical analyses were carried out using SPSS for Windows, version 17.0 (SPSS Inc., Chicago, IL), and a *P*-value of less than 0.05 was considered to indicate statistical significance.

## RESULTS

**Figure 1:**
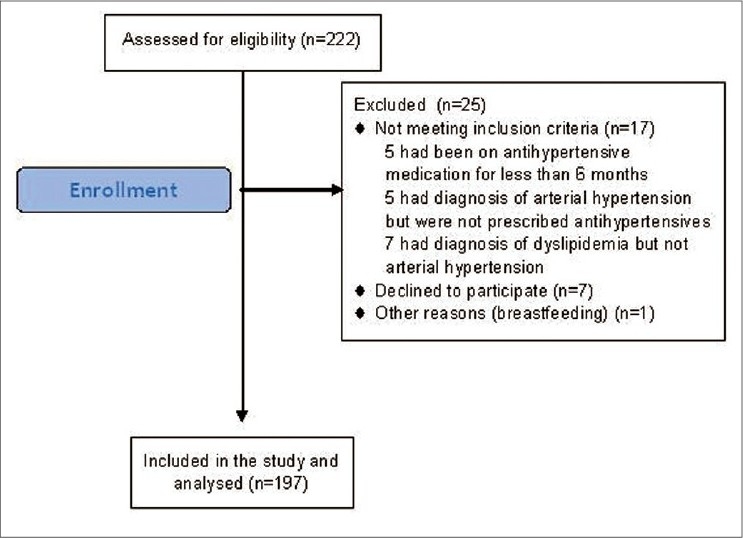
Diagram of patient enrollment.

A total of 222 patients attended the medical clinic during the recruitment period (from July 2009 to September 2009), and all were assessed for eligibility. Of these, 17 were excluded from the study because they did not meet the inclusion criteria, 1 was excluded because of breastfeeding, and 7 were excluded because they declined to participate (they did not sign the informed consent). Thus, a total of 197 (89%) hypertensive patients met the inclusion criteria and consented to participate [Figure 1].

The overall mean age of the included patients was 60 ± 12 years, 40.1% being male and 59.9% female [Table 1]. Among these patients, 153 (77.7%) had neither diabetes nor CKD and were considered to have a target BP of <140/90 mmHg, whereas the remaining 44 (22.3%) had either diabetes and/or CKD and were considered to be controlled with a BP of <130/80 mmHg. Most patients were long-term hypertensives, with 74.1% (146/197) of patients taking antihypertensive medications for at least 5 years. Only four patients have been prescribed hypertensive medication for the first time in the preceding 12 months. Overall, only 48.2% of patients were considered to be highly adherent to antihypertensive medication. Other demographic and clinical characteristics, as well as results obtained in the structured interview are represented in Table 1. All variables listed in Table 1 were covariates in the analyses.

**Table 1:** Patients demographics and clinical characteristics and data collected in the structured interview (n = 197)

Demographic/clinical characteristics	Values
Gender, n (%)	
Male	79 (40.1)
Female	118 (59.9)
Age, mean (SD)	59.5 (11.7)
Body mass index (kg/m2), mean (SD)	29.4 (4.8)
Married, n (%)	160 (81.2)
Education, n (%)	
Illiterate	10 (5.1)
Elementary schooling	156 (79.2
High schooling	20 (10.2)
University education	11 (5.6)
Current smoker, n (%)	17 (8.6)
Comorbid conditions, n (%)	
Cerebrovascular disease	26 (13.2)
Chronic kidney disease	11 (5.6)
Diabetes	36 (18.3)
Heart failure	1 (0.5)
Ischemic heart disease	5 (2.5)
Myocardial infarction	3 (1.5)
Left ventricular hypertrophy	5 (2.5)
Dyslipidemia	148 (75.1)
Metabolic syndrome	4 (2.0)
Obesity (body mass index ≥ 30 kg/m2)	81 (41.1)
Number of years in antihypertensive drug treatment, mean (SD)	9.8 (7.7)
Low self-reported medication adherence, score ≥ 3, n (%)	102 (51.8)
Knowledge of target BP values, n (%)	117 (59.4)
Knowledge of hypertension risks, n (%)	108 (54.8)
Knowledge of drug indications, n (%)	139 (70.6)
Regular monitoring of BP, n (%)	139 (70.6)
Reported side effects, n (%)	45 (22.8)

Abbreviations: BP, Blood pressure; SD, standard deviation.

Overall, the mean systolic BP of the 197 hypertensive patients included in our analysis was 141.8 ± 16.5 mmHg, and the mean diastolic BP was 85.8 ± 11.80 mmHg, with 33.0% (65/197) patients attaining controlled BP according to JNC 7 guidelines. Among patients with neither diabetes nor CKD, 39.2% had their BP controlled, whereas among patients with one or both of these pathologies this figure was 11.4% (*P* = 0.009). In all, 37.1% (73/197) patients attained BP values <140/90 mmHg.

Forward:LR logistic regression revealed that the covariates medication adherence (OR, 4.8; 95% CI, 2.4–9.5; *P* < 0.001), marital status (OR, 5.3; 95% CI, 1.7–16.4; *P* < 0.004), and diabetes (OR, 4.4; 95% CI, 1.4–13.5; *P* < 0.011) were the independent variables that significantly influenced BP control. Using the logistic regression coefficients (ln(OR)) for those covariates, the following significant logistic regression model (*G*^2^ (3) = 46.054; *P* < 0.001; *X*^2^ _HL_(4) = 4.152; *P* = 0.386; R^2^ _CS_ = 0.208; R^2^ _N_ = 0.290; R^2^ _MF_ = 0.184, where G^2^ is the likelihood-ratio goodness-of-fit statistic, *X*^2^ _HL_ is the Hosmer and Lemeshow statistic, R^2^ _CS_ is the Cox and Snell R square, R^2^ _N_ is the Nagelkerke R square, and R^2^ _MF_ is the McFadden R square) was obtained:

$$\mathrm{Pb}\;=\;\frac{e\left(-0.436+1.562\mathrm{ADH}+1.670\mathrm{MS}+1.472\mathrm{DIAB}\right)}{1+e\left(-0.436+1.56\mathrm{ADH}+1.670\mathrm{MS}+1.472\mathrm{DIAB}\right)}$$

(1) where Pb is the probability of a given patient to have uncontrolled BP, ADH is medication adherence (0, adherent; 1, nonadherent), MS is marital status (0, married; 1, single, widowed, divorced, or separated), and DIAB is diabetes (0, without diabetes; 1, with diabetes).

This model has acceptable sensitivity (77.3%) and specificity (63.1%), as well as an acceptable discrimination power (area under ROC curve = 0.764; *P* < 0.001).

Of the three covariates figuring in Eq. (1), medication adherence is the independent variable more likely to be favorably influenced by a health care professional team in order to enhance BP control. Thus, we determined the covariates that significantly influence the dependent variable medication adherence. Forward:LR logistic regression revealed that knowledge of target BP (OR, 3.7; 95% CI, 1.9–7.4; *P* < 0.001), reporting of drug side effects (OR, 3.7; 95% CI, 1.6–8.3; *P* < 0.002), measuring BP regularly (OR, 2.5; 95% CI, 1.2–5.2; *P* < 0.015), knowledge of drug indications (OR, 2.4; 95% CI, 1.1–5.2; *P* < 0.021), and knowledge of hypertension risks (OR, 2.1; 95% CI, 1.1–4.2; *P* < 0.026) were the independent variables that significantly influences medication adherence. Using the logistic regression coefficients (ln(OR)) for those covariates, the following significant logistic regression model (*G*^2^ (5) = 54.446; *P* < 0.001; *X*^2^ _HL_ (7) = 2.448; *P* = 0.931; R^2^ _CS_ = 0.241; R^2^ _N_ = 0.322; R^2^ _MF_ = 0.200) was obtained:

$$\mathrm{Pb}\;=\;\frac{e\left(-1.546+1.307\mathrm{TBP}+1.296\mathrm{SE}+0.906\mathrm{BPR}+0.89\mathrm{DI}+0.759\mathrm{HTR}\right)}{1+e\left(-1.546+1.307\mathrm{TBP}+1.296\mathrm{SE}+0.906\mathrm{BPR}+0.89\mathrm{DI}+0.759\mathrm{HTR}\right)}$$

(2) where Pb is the probability of a given patient to be nonadherent to medication, TBP, knows target BP (0, yes; 1, no); SE, reports side effects (0, no; 1, yes); BPR, measures BP regularly (0, yes; 1, no); DI, knows drug indications (0, yes; 1, no); and HTR, knows hypertension risks (0, yes; 1, no).

This model also revealed to have acceptable sensitivity (71.6%) and specificity (72.6%), as well as an acceptable discrimination power (area under ROC curve = 0.788; *P* < 0.001).

All independent variables figuring in Eq. (2) are amenable to improvement by team-based health care professionals’ interventions in order to increase medication adherence and, thereby, BP control.

## DISCUSSION

The results presented in this study describe some demographic and clinical characteristics of hypertensive patients attending the medical consultation of hypertension/dyslipidemia in a university teaching hospital located in the Eastern Central Region of Portugal, focusing on the level of hypertension control and antihypertensive medication adherence. According to a survey conducted in 2003,[1] of the total number of hypertensives in the Central Region of Portugal prescribed with antihypertensives, only 26.1% had their BP measurements <140/90 mmHg, which is significantly smaller than the 37.1% obtained in our study. This difference possibly points to an improved current care of the hypertensives included in our study when compared to those included in the abovementioned survey. Increased awareness of hypertension and the importance of lower BP may have prompted Portuguese providers and patients to treat high BP more aggressively, especially after the publication of the JNC 7 report in 2003. The issuing, in 03/31/2004, of the legal document “Guidelines to detect, treat and control arterial hypertension”,[6] by the Department of Health of the Portuguese Government, definitely contributed to an evidence-based approach to the prevention, detection, evaluation, and treatment of high BP. These guidelines have many similarities with the JNC 7 report and were subsequently updated, in 2006, by the Portuguese Society of Hypertension.[7] Till now, there was no prospective data about the percentage of treated hypertensives in clinical practice, in this Portuguese region, with their BP controlled according to the JNC 7 guidelines. Our study revealed that 33.0% of hypertensive patients had their BP controlled according to those guidelines, and that there was a significantly greater BP control (*P* = 0.009) in patients without diabetes or CKD (39.2%) when compared to patients with diabetes and/or CKD (11.4%). To the best of our knowledge, this prospective study presents for the first time the percentage of treated and controlled hypertensives, according to the JNC 7 guidelines, in a Portuguese subpopulation. It should be noted that the reported levels of BP control can vary greatly depending on the study population, methods, and time frame.[8,9] In one study, on the basis of data from the US National Health and Nutrition Examination Survey 2003–2004, the BP control rate (to <140/90 mmHg) was 56.6% in treated hypertensives, and 37.5% in treated hypertensive persons with diabetes mellitus (for whom the goal BP is <130/80 mmHg).[10] In a regional survey performed in the middle-West of France and involving 1050 treated hypertensives, Ragot *et al*., reported that 39% of patients had BP figures <140/90 mmHg and only 13% of the diabetic population were normalized according to the international recommendations (<130/80 mmHg).[11] In a more recent retrospective observational study conducted in the United States, Jackson *et al*.[9] reported a BP control of 49.3% in an after-JNC 7 cohort. In this cohort, a significantly higher percentage of nondiabetic patients achieved BP control compared with those with comorbid diabetes (60.9% vs. 29.4%). Similarly, Andros *et al*.[12] conducted a retrospective observational study of BP control in an insured diabetic population, obtaining a BP control rate (defined by JNC 7) of 28%, similar to the 29.4% obtained by Jackson *et al*.[9] The results obtained in our study are similar to those reported by Ragot *et al*., in a middle-West French treated hypertensive population, for both patients without diabetes and CKD and patients with these pathologies.

The logistic regression analyses of the study population revealed that the covariates medication adherence, marital status and diabetes significantly influence BP control, such that the probability of a nonadherent, unwed, diabetic patient to have uncontrolled hypertension is 98.6%. At the other end, the probability of an adherent, married, nondiabetic patient to have uncontrolled hypertension is only 39.3%. Surely, there are more unstudied independent variables that significantly influence BP control (e.g., prescribed antihypertensive medication). For example, the possible existence of clinical inertia and undertreatment must be analyzed in patients with diabetes or CKD. However, sensitivity, specificity, and ROC curve analysis revealed an acceptable model performance.

Of the three covariates significantly influencing BP control, the variable most likely to be favorably influenced by team-based health care professionals’ interventions is medication adherence. In patients with hypertension, medication nonadherence is a significant, often unrecognized, risk factor that contributes to poor BP control, thereby contributing to the development of further vascular disorders such as heart failure, coronary heart disease, renal insufficiency, and stroke.[13] Antihypertensive medication adherence rates have differed widely depending on the population studied, and it is estimated to range between 50% and 70% in patients with treated hypertension.[14–16] Therefore, the percentage of antihypertensive medication adherence found in our study is largely within the range reported in the literature.[17,18] The importance of improving adherence to antihypertensive medication has been addressed by JNC 7, and emphasis has been put on the role of all health care professionals, including pharmacists, to improve adherence to the treatment.[19]

Logistic regression revealed that knowledge of target BP values, the presence of drug side effects, measuring BP regularly, knowledge of drug indications, and knowledge of hypertension risks are the independent variables that significantly influence medication adherence. According to our logistic regression model (Eq. (2)), the probability of a hypertensive patient ignoring target BP values, drug indications and hypertension risks, reporting drug side effects and not monitoring BP regularly to be nonadherent is 97.4%. On the contrary, the probability of a hypertensive patient knowing the target BP values, drug indications and hypertension risks, not presenting drug side effects and monitoring BP regularly to be nonadherent is only 17.6%.

Although a significant percentage (70.6%) of patients reported to measure BP regularly, only (*P* = 0.020) 59.4% were aware of their target BP figures (systolic and diastolic). Self and/or regular BP measurement is useful for the assessment of the treatment effects by the doctor and is valuable for the patients in improving the management of their high BP. However, as many patients (40.6%) do not know their target BP values they cannot accurately report whether it is controlled. Therefore, a major implication of our study is the need for education of patients to their target BP figures so that they can correctly identify whether it is elevated or controlled. Likewise, our findings also suggest the need to improve patient awareness of the cardiovascular risks of hypertension and of the therapeutic indications and usefulness of antihypertensives. It is worth noting that even though these patients have had hypertension for a long period (average number of years in antihypertensive drug treatment was 9.8 ± 7.7 years), their knowledge is inadequate. Recent research points to the need to improve hypertension knowledge and awareness in order to increase medication adherence and BP control.[20,21] Continuing attention should be given to side effects of antihypertensives, because they are one of the most important causes of nonadherence.[22] Adverse events during antihypertensive treatment are not entirely avoidable because they are partly psychological and are also reported during administration of placebo.[22] However, great effort should be dedicated to limitation of drug-related side effects and preservation of the quality of life either by switching treatment from the responsible drug to another agent or by avoiding unnecessary increases of the dose by using combination therapy.[23] Discussion of patients’ side effects and concerns must also be encouraged.

Several features of our study deserve further comment. To our knowledge, this is the first Portuguese study examining barriers to BP control in treated hypertensive outpatients in the Eastern Central Region of Portugal. The study may be relevant to similar Caucasian populations in Portugal and other European countries. Other strengths were the high response rate and the 96.6% completion of questionnaires. The limitations are the size of the study, and the evaluation of BP control based on the measurements performed in one single medical appointment. These BP measurements may or may not be representative of the adequacy of BP control in hypertensive patients. We also were unable to obtain objective measurements of patient compliance (e.g., drug level in biologic fluids, biologic markers, and direct patient observation) or to assess the attitudes of the physicians and nurses toward patients. Behavioral models suggest that the most effective therapy prescribed by the most careful physician will control hypertension only if the patient is motivated to take the medication as directed.[19] Motivation improves when patients have positive experiences with, and trust in, their health care professional team.[19] Moreover, a limited number of covariates were analyzed. Undertreatment and clinical inertia are reported causes of uncontrolled BP[24,25] that were not evaluated in this cross-sectional study. Further, for some subgroup analyses, there were small cell sizes and the analyses must be considered exploratory in nature.

In conclusion, this study provides a framework for identifying hypertensive patients who are at high risk of poor BP control and since many of the identified factors are modifiable, they signal opportunities to improve BP control in clinical practice. Poor medication adherence and patient unawareness about target BP values, hypertension risks and antihypertensive drug indications, as well as the presence of drug side effects and lack of regular BP monitoring should be considered as possible underlying causes of inadequately controlled BP and must be addressed in any intervention aimed to improve BP control. Strategies to improve medication adherence must be reinforced. Self-monitoring with validated BP devices should be encouraged. Patients with poor knowledge of the goal of their hypertension therapy should be informed about their target BP, to enable them to participate more fully in their own management. To achieve the crucial objective of improving health by controlling high BP, it is important to fully understand the current status of patient knowledge, awareness, and attitudes with respect to hypertension, medication, and lifestyles. It is necessary to understand these patient factors to develop effective strategies and team-based health care professionals’ interventions that enroll the patient as a participant in the management of his hypertension.
